# Layer by Layer Antimicrobial Coatings Based on Nafion, Lysozyme, and Chitosan

**DOI:** 10.3390/nano9111563

**Published:** 2019-11-04

**Authors:** Ella N. Gibbons, Charis Winder, Elliot Barron, Diogo Fernandes, Marta J. Krysmann, Antonios Kelarakis, Adam V. S. Parry, Stephen G. Yeates

**Affiliations:** 1 School of Pharmacy and Biomedical Sciences, University of Central Lancashire, Preston PR1 2HE, UK; 2 UCLan Research Centre for Smart Materials, School of Physical Sciences and Computing, University of Central Lancashire, Preston PR1 2HE, UK; 3 Malvern Panalytical, Enigma Business Park, Grovewood Road, Malvern, Worcestershire WR14 1XZ, UK; 4 School of Chemistry, University of Manchester, Manchester M13 9PL, UK

**Keywords:** antimicrobial, layer-by-layer, coatings, Nafion, multilayers

## Abstract

The study focuses on the development of a new family of layer-by-layer coatings comprising Nafion, lysozyme and chitosan to address challenges related to microbial contamination. Circular dichroism was employed to gain insights on the interactions of the building blocks at the molecular level. Quartz crystal microbalance tests were used to monitor in real time the build-up of multilayer coatings, while atomic force microscopy, contact angle and surface zeta potential measurements were performed to assess the surface characteristics of the multilayer assemblies. Remarkably, the nanocoated surfaces show almost 100% reduction in the population of both *Escherichia coli* and *Staphylococcus aureus*. The study suggests that Nafion based synergistic platforms can offer an effective line of defence against bacteria, facilitating antimicrobial mechanisms that go beyond the concept of exclusion zone.

## 1. Introduction

The quest for advanced antimicrobial materials is driven by the large diversity of remarkably adaptable pathogens coupled with the alarming evolution of drug resistant strains that cause serious infections to humans and the ecosystem [1,2,3,4]. Despite preventive measures and increased public awareness, contiguous bacterial colonies are found on a variety of surfaces such as foodservice equipment, water distribution pipelines, swimming pools, lakes, rivers, public transport vehicles, toilets, door handles, home appliances, and air-conditioning filters. At the same time, bloodstream infections originating from catheters, implants, and surgical tools result in enormous costs for the healthcare system [5].

Layer by layer (LbL) assemblies, based on the alternated adsorption of oppositely charged molecules or nanoparticles, is a versatile approach that affords control at the nanoscale level, generating stable and robust coatings [6,7,8]. Based on those principles, a wide range of LbL antimicrobial coatings comprising polymers, nanoparticles, enzymes, peptides, biological molecules, and antibiotics as building units has been reported [9]. Their antimicrobial performance relies on bioadhesion resistance, contact-killing, release-killing, or a combination of those mechanisms [9].

Along those lines, the positively charged amidated ponericin G1, a strong antimicrobial against *Staphylococcus aureus* (*S. aureus*), was incorporated to hydrolytically degradable LbL coatings based on poly (b-amino esters). The peptide was released from the film in a controlled manner and was effective in inhibiting bacteria attachment, thus demonstrating significant potential for implant materials and bandages [10]. In a contact-killing demonstration, LbL assemblies comprising poly (allylamine hydrochloride) and poly (sodium 4-styrene sulfonate) showed sufficient density of mobile cations that endowed significant antimicrobial activity [11]. In an anti-adhesion strategy, poly (L-lysine)/poly (L-glutamic acid) multilayers with the top bilayers bearing the pegylated polyanion drastically suppressed the adsorption of *Escherichia coli* (*E. coli*) [12].

In this work, we focus on LbL assemblies comprising two naturally occurring antimicrobials, namely lysozyme [13] and chitosan [14] along with Nafion, a synthetic ionomer with a robust, Teflon-like backbone bearing hydrophilic sulphonic acid groups. As a direct consequence of its chemical composition, Nafion forms proton exchange membranes with supreme structural and chemical stability that set the benchmark for fuel cell applications [15]. Moreover, Nafion has been shown to possess an exclusion zone against bacterial growth, an effect that has been attributed to repulsive forces between its negatively charged surface and the similarly charged cell membranes [16,17]. In this work, we demonstrate that, although the surface charges of Nafion have been neutralised (if not overcompensated) by the adsorption of positively charged molecules, the coatings show remarkable antimicrobial activity against *E. coli* and *S. aureus*. In that sense, our work paves the way for the development of a new family of Nafion-based nanostructured coatings with enhanced antimicrobial performance that do not necessarily rely on exclusion zone effects.

## 2. Materials and Methods

### 2.1. Materials

Nafion (DE 1021) (Chemours Company, Wilmington, DE, USA) with a total H^+^ exchange capacity of 1.1 mequiv/g was obtained as a 10 wt% dispersion in water (Ion-Power) or as a 15 wt% dispersion in a mixture of low aliphatic alcohols (3-propanol, ethanol, and others) and water (Ion Power). Lysozyme (Buchs, Switzerland) from chicken eggs (106 U/mg) and medium molecular weight chitosan (Milwaukee, WI, USA) were obtained from Sigma Aldrich (Dorset, U.K). Chitosan was dispersed in water containing 0.1 wt% acetic acid.

### 2.2. Quartz Crystal Microbalance with Dissipation Monitoring (QCM-D)

The quartz crystal microbalance with dissipation monitoring (QCM-D) tests were performed using a Q-sense E1 unit (Biolin Scientific, Stockholm, Sweden) equipped with a Peltier-controlled flow cell (flow rate was set at 0.2 mL/min) with temperature accuracy of 0.02 °C. Au-modified crystals with a fundamental resonance frequency close to 5 MHz and diameter 150 nm were spin-coated (by depositing a drop of a 0.5 wt% Nafion solution in ethanol) and were then left at room temperature for at least seven days. All measurements included an initial equilibrium step of the crystal in the air to determine the fundamental resonant frequency, followed by an equilibrium step under constant flow of water to establish the baseline of the hydrated surface.

On the basis of Sauerbrey relation: Δ*m* = −(*C*/*N)* Δ*f*, deposition of a uniform layer with mass Δm reduces the resonant frequency of the crystal by Δ*f*, where *N* is the overtone number (herein all values reported refer to *N* = 3) and *C* is the integrated crystal sensitivity that depends upon the intrinsic properties and the thickness of the crystal [18]. The dissipation factor *D* is defined as *D* = *E_d_*/(2π*E_s_*), where *E_d_* is the energy dissipated during one period of oscillation and *E_s_* is the energy stored in the system [19].

### 2.3. Contact Angle Measurements

The contact angle of distilled water droplets (5 μL) deposited on the coated quartz crystals was determined by means of an OptoSigma (OptoSigma Corp., Santa Ana, CA, USA) optical tensiometer using the standard sessile drop technique (Digidropmeter, GBX). The photos were captured 20 s following the deposition of the droplets. A minimum of five spots on each specimen were measured.

### 2.4. Circular Dichroism (CD) Spectrometry

Lysozyme solutions in the presence and absence of Nafion were inserted to a quartz cuvette of 0.1 cm light path length and their circular dichroism (CD) spectra at 25 °C were collected using a Jasco J-815 CD spectropolarimeter (Jasco, Tokyo, Japan). Each spectrum was collected for five accumulations, with a scanning range from 260 to 180 nm, a band width of 2 nm, data pitch of 0.5 nm, digital integration time of 1 s, and a scanning speed of 100 nm/min. Values for the baseline (measured without a cuvette) and the blank solutions (ultrapure water) were subtracted from test values. The CD spectra in terms of α-helix, β-sheet, and random structures were analysed using DichroWeb [20]. All samples had total concentration of 0.01 mg/mL, but varying f_Lys_ values, where f_Lys_ stands for weight of lysozyme/weight of Nafion.

### 2.5. Atomic Force Microscopy (AFM)

The samples were mounted on magnetic sample holders for AFM tests. The measurements were performed on a Park XE-100 (Parksystems, South Korea) in non-contact mode using a cantilever with spring constant of approximately 40 N/m. Images were taken with a 512 by 512 pixel resolution at a scan rate of between 0.2 and 0.5 Hz.

### 2.6. Surface Zeta Potential (ζ_surface_)

(Naf/Lys)_6_, (Naf/Chi)_6_, and (Naf/Lys/Naf/Chi)_2_ coatings were deposited on Nafion-precoated aluminium foil and polypropylene substrates via standard dip-coating protocols. ζ_surface_ measurements were recorded at 25 °C in a surface zeta cell apparatus, at a forward angle of detection (13°) on a Zetasizer Nano ZS, (Malvern Panalytical, Malvern, UK). Data were recorded at different displacement distances from the surface which then allow for the surface zeta potential to be calculated, according to the equation below:

ζ_surface_ = −ζ_intercept_ + ζ_tracer particles_ (1)

where ζ_intercept_ is the zeta potential at displacement 0 from the surface, calculated from a linear regression fit. Four repeat measurements were recorded at each displacement of 1.25 µm from the previous point, with a total of four displacement points.

Polystyrene beads (DTS1235, −42 mV ± 10%) were used as the tracer particles and water was selected as the dispersant. The measured electrophoretic mobilities (U_E_) were converted into ζ values assuming Smoluchowski approximation *F*(ka) = 1.5 for Henry’s equation *U*_E_ = 2εζ*F*(ka)/3η, where ε, η are the dielectric constant and the viscosity of the dispersant, respectively [21].

### 2.7. Antimicrobial Testing

A. Culturing Method. 250 mL Erlenmeyer flasks containing 25 mL nutrient broth were inoculated with a single loop of bacteria and incubated for 24 h in a SciQuip Incu-Shake MIDI orbital shaker (SciQuip Ltd, Newtown, Wem, Shropshire, UK) set to 200 rpm at 37 °C. Cultures were centrifuged at 4000 rpm for 10 min. Subsequently, the supernatant was discarded, 20 mL of 1/4 strength Ringer’s solution was added, and the tubes were vortexed.

Tubes were centrifuged for a further 10 min at 4000 rpm and supernatant again discarded. 2 mL of ¼ strength Ringer’s solution was added and tubes vortexed a final time. Resuspended cultures were diluted in nutrient broth to obtain an absorbance reading equivalent to a 0.5 McFarlane standard as recorded by Biochrom WPA S800 visible spectrophotometer (Biochrom Ltd, Cambridge, UK).

B. Disk testing method. Disk testing method was adapted from the literature [22]. Each disk was assigned to one of the twelve wells using a random number generator. Each of these wells was lined with sterile aluminium foil, for ease of removal of disks and prevention of bacterial run-off. An additional well was filled with 1mL sterile distilled deionized water to prevent dehydration of samples.

200 µL bacterial culture was added to each disk and was incubated for 20 h at 37 °C. Following incubation, each disk was transferred along with the foil into 9.8 mL 1/4 strength Ringer’s solution and sonicated for 10 min. Sonicated solutions were serially diluted 100 µL sample solution into 900 µL 1/4 strength Ringer’s solution. A 100 µL respective sample was used to spread onto each nutrient agar plate, each dilution being plated in triplicate. Plates were incubated for 20 h at 37 °C, then counted for colonies. For each type of coating five crystals were tested and the average values were determined.

## 3. Results

**Figure 1:**
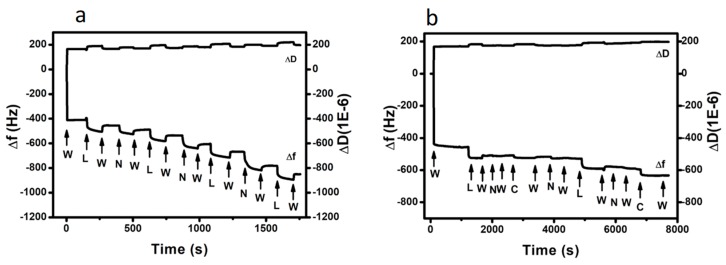
QCM-D sensograms at 25 °C monitoring the build-up of; (**a**) (Naf/Lys)_4_ and (**b**) (Naf/Lys/Naf/Chi)_2_. The letters “W”, “N”, “L”, and “C” signify the injection of water, Nafion, lysozyme, and chitosan solution, respectively.

The QCM-D sensogram shown in Figure 1a describes the build-up of four Nafion/lysozyme bilayers on a Nafion precoated crystal resonator. We note that Nafion combines a hydrophobic backbone with hydrophilic perfluoroether side chains terminated with sulphonic acid groups and, thus, it undergoes microphase separation into polar and nonpolar domains. In particular, Nafion is described as a network of parallel water-filled channels held in place by the cross-linking action of the crystalline domains [23].

As seen in Figure 1a, injection of water results in a pronounced drop of the oscillating frequency coupled with a corresponding increase in the dissipation factor, indicating significant swelling of the ionic channels. The baseline recorded for the hydrated Nafion membrane remains stable, eliminating the possibility of even minor dissolution under water flow. Upon water exposure, Nafion’s surface is reorganised with the sulphonic groups to be turned into the aqueous phase, a mechanism that gives rise to a large water contact angle hysteresis [24]. Owing to its amphiphilic nature Nafion has been shown to bind not only with charged polymers such as poly (oxyethylene) [25] and poly (oxypropylene) based diamines [26], but also with non-ionic surfactants via hydrogen bonding, hydrophobic interactions [27,28,29].

As evident by the significant drop in Δ*f* (Figure 1a), lysozyme (at pH = 6.2), a ubiquitous enzyme widely used as food preservative, is massively adsorbed on the hydrated Nafion film. Given that lysozyme bears positive charges within the pH range 1–11 [30], it is strongly attracted to the negatively charged Nafion, so that subsequent rinsing with water removes only a limited amount of weakly bound lysozyme molecules. The Nafion/lysozyme deposition cycle was repeated for three more times in an identical fashion to generate an ultrathin LbL membrane denoted hereafter as (Naf/Lys)_4_, while the deposition of two further bilayers led to (Naf/Lys)_6_. Likewise, the (Naf/Chi)_6_ LbL coating was assembled using chitosan, an aminopolysaccharide biopolymer with a broad antimicrobial spectrum that is extensively used to prevent bacterial contamination in food and drug packaging, as the positively charged layer. The QCM-D sensogram in Figure 1b describes the formation of a (Naf/Lys/Naf/Chi)_2_ assembly as a three-component coating that relies on the attractive Nafion/lysozyme and Nafion/chitosan forces. The action of those attractive forces is further confirmed by the spontaneous precipitation that takes place upon mixing 0.1 wt% Nafion with either 0.1 wt% lysozyme (pH = 6.2) or 0.1 wt% chitosan. Note that the injection of chitosan results in a rather limited drop in Δf compared to lysozyme, presumably due to enhanced steric hindrance.

**Figure 2:**
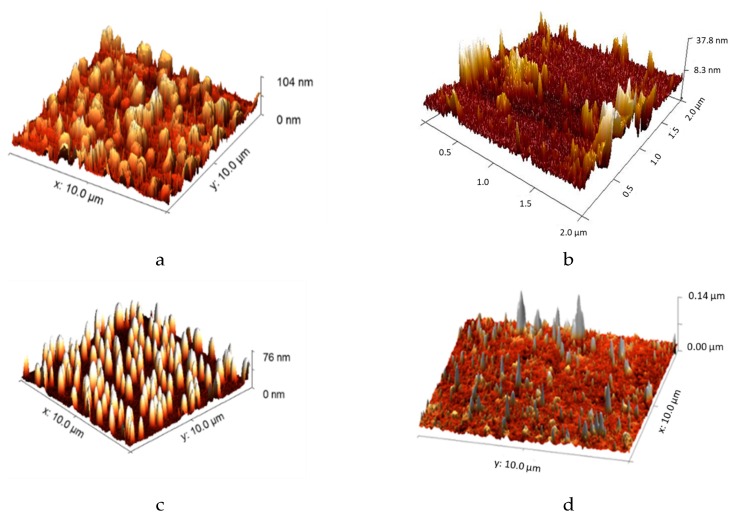
AFM images of the: (**a**) Nafion, (**b**) (Naf/Lys)_6_, (**c**) (Naf/Chi)_6_, and (**d**) (Naf/Lys/Naf/Chi)_2_ coatings deposited on quartz crystal microbalance with dissipation monitoring (QCM-D) crystals.

Figure 2 displays AFM images of the Nafion, (Naf/Lys)_6_, (Naf/Chi)_6_, and (Naf/Lys/Naf/Chi)_2_ coatings. It has been demonstrated that lysozyme adsorbed on a solid surface undergoes pronounced conformational reorganization driven by hydrophobic-hydrophobic interactions, ultimately resulting in the formation of aggregates that diffuse on the surface [31]. Chitosan molecules on a solid substrate follow similar clustering/agglomeration patterns, ultimately adopting significant levels of surface roughness [32]. At the same time, the topological characteristics of Nafion mirror the microphase separation of the bulk and are largely dependent on the relative humidity and the hydration levels [33].

**Figure 3:**
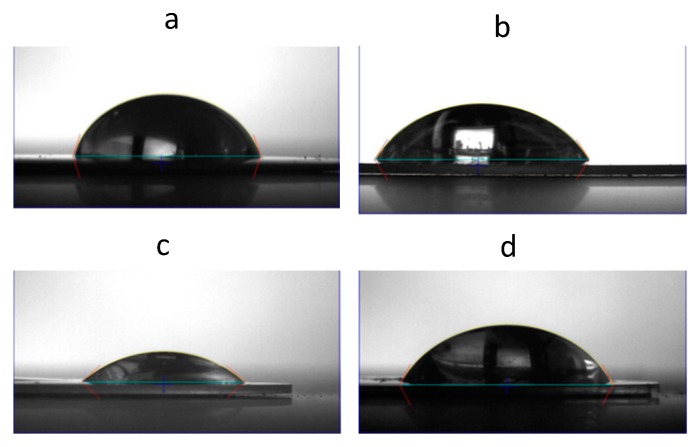
Water contact angles of: (**a**) Nafion, (**b**) (Naf/Lys)_6_, (**c**) (Naf/Chi)_6_, and (**d**) (Naf/Lys/Naf/Chi)_2_ coatings deposited on QCM-D crystals.

As shown in Figure 3, the water contact angles for (Naf/Lys)_6_, (Naf/Chi)_6_, and (Naf/Lys/Naf/Chi)_2_, were found to be 45.3°, 59.0°, and 65.1°, respectively, compared to 73.3° for a Nafion coated surface. It is generally accepted that hydrophobic surfaces are desirable for antimicrobial applications, however there is evidence to suggest that intermediate contact angles, as those found in the present systems, might also be compatible with advanced antimicrobial behaviour [34]. The above coatings were applied to polystyrene surfaces without compromising their optical transparency (Figure S1a), although the nanocoated surfaces showed enhanced UV-vis absorbance (Figure S1b). The development of transparent, yet UV blocking packaging materials with advanced antimicrobial properties are of supreme importance in the food industry and the coatings disclosed here point to this direction.

**Figure 4:**
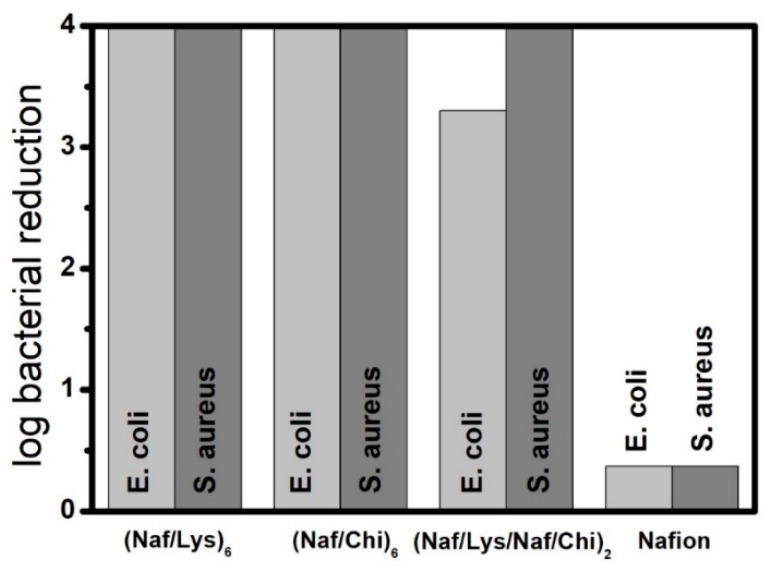
Reduction (in log scale) of the population of *E. coli* and *S. aureus* cultures exposed to (Naf/Lys)_6_, (Naf/Chi)_6_, (Naf/Lys/Naf/Chi)_2_, and Nafion coated QCM-D crystals. (For each type of coating five crystals were tested and the average values are shown in this figure).

As shown in Figure 4, the (Naf/Lys)_6_, (Naf/Chi)_6_, and (Naf/Lys/Naf/Chi)_2_ coatings inhibit *E. coli* growth by 99.99%, 99.99%, and 99.95%, respectively, compared to 57.7% for the Nafion coated crystal. Moreover, the (Naf/Lys)_6_, (Naf/Chi)_6_, and (Naf/Lys/Naf/Chi)_2_ coatings all inhibit *S. aureus* growth by 99.99%, compared to 57.1% for the Nafion coated crystal. For reference, (Naf/Chi)_3_ and (Naf/Lys/Naf/Chi)_1_ coatings reduce *E. coli* by 74.6% and 88.5%, respectively, and inhibit *S. aureus* growth by 99.9% and 83.4%, respectively (Figure S2). 

**Figure 5:**
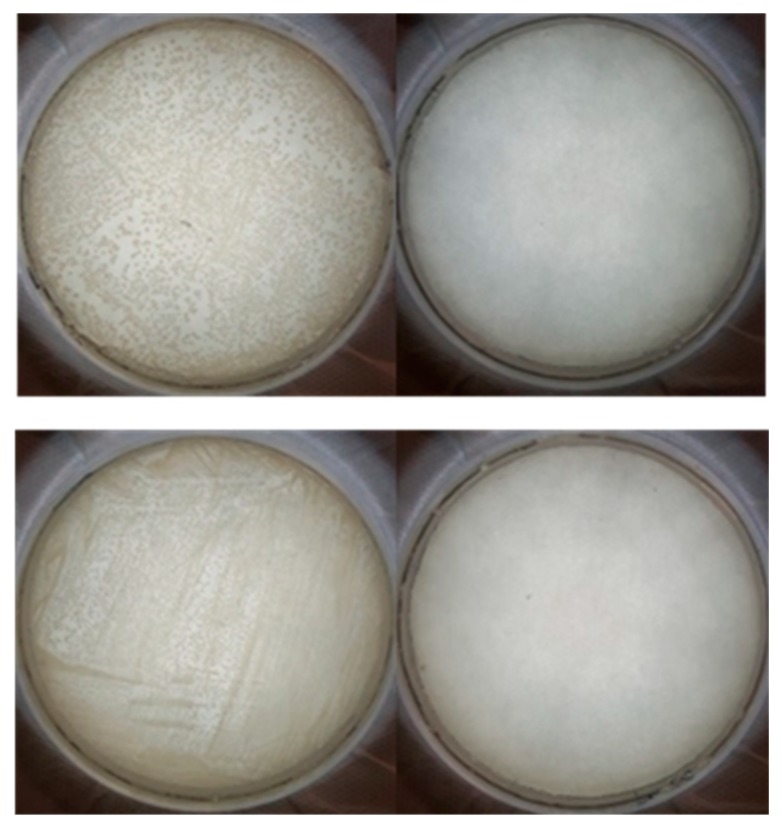
Photos of the petri dishes containing *E. coli* (upper photos) and *S. aureus* (lower photos) cultures. The petri dishes on the left have been exposed to uncoated QCM-D crystals, while those on the right have been exposed to (Naf/Lys)_6_ coated discs, under otherwise identical conditions.

The photos displayed in Figure 5 clearly depict the significant advantages of the nanocoatings disclosed here. When *E. coli* and *S. aureus* cultures are exposed to uncoated QCM-D crystals (blank samples), no antimicrobial effect is observed. In contrast, when the same *E. coli* and *S. aureus* cultures are exposed to, otherwise identical, (Naf/Lys)_6_, (Naf/Chi)_6_, and (Naf/Lys/Naf/Chi)_2_ coated QCM-D crystals, virtually all bacteria appear to be eliminated. Such a remarkable antimicrobial performance might reflect the synergistic effect of the contact-killing behaviour of lysozyme and chitosan, the bacteria-repelling behaviour of Nafion combined with contributions arising from surface roughness and wettability. Standard agar diffusion tests indicated the absence of bacteria inhibition zones around the coated crystals, confirming that the building blocks of the LbL assemblies are firmly fixed and do not diffuse into the agar.

The antibacterial performance of lysozyme stems from its enzymatic activity to cleave 1,4 beta-linkages between N-acetylmuramic acid and N-acetyl-D-glucosamine that triggers peptidoglycan hydrolysis and, ultimately, cell lysis. Evidently, this mechanism is less effective for Gram-negative bacteria whose protective outer membranes prevents access to the enzyme [35]. Adjusting the pH of the lysozyme solution to 4 and 9 decreases the efficiency of (Naf/Lys)_6_ against *E. coli* but leaves its ability to combat *S. aureus* essentially intact (Figure S2). It is noted that the lytic activity of lysozyme has been found to exhibit a maximum at pH 6.2 over a broad range of ionic strengths [36].

**Figure 6:**
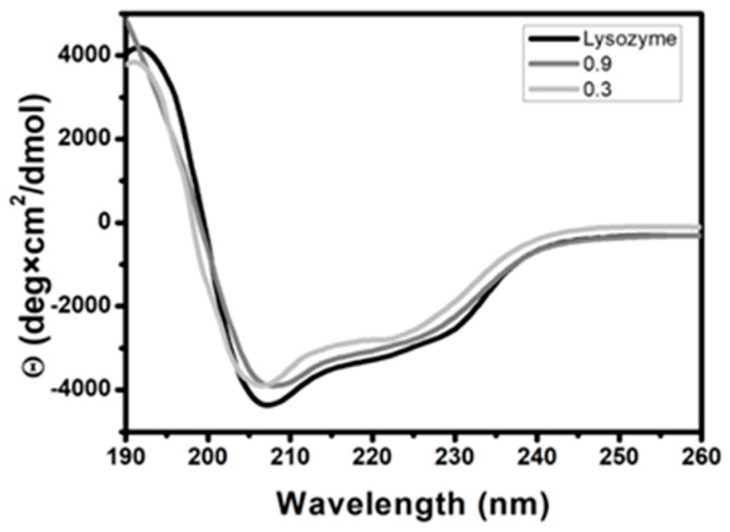
Circular dichroism (CD) spectra of aqueous solutions containing: Lysozyme and lysozyme/Nafion mixtures at f_Lys_ = 0.9 and f_Lys_ = 0.3 at 25 °C.

This supreme antimicrobial performance of lysozyme is only encountered on the condition that it’s secondary structure is well preserved [37]. The CD spectrum of lysozyme shown in Figure 6 is dominated by two negative bands at 208 and 222 nm and suggests the presence of 71% α-helix and 10% β-sheet, consistent with data published previously [38]. Upon mixing with Nafion at f_Lys_ = 0.9 and f_Lys_ = 0.3, the secondary structure of lysozyme is modified to a small extent, given that the α-helix content decreases to 64% and 50% and the β-sheet content increases to 16% and 23%, respectively.

## 4. Discussion

A number of LbL assemblies employing lysozyme as a key antimicrobial ingredient has been reported in the literature. To that end, LbL assemblies comprising lysozyme and pectin deposited on cellulose mats were shown to induce a clear zone of bacterial inhibition, the more so when lysozyme is present at the outermost layer [39]. In a similar manner, lysozyme and gold nanoparticles were deposited on cellulose nanofibrous mats improving their antimicrobial performance against Gram-positive as well as Gram-negative model bacteria [40]. In addition, mechanically robust LbL membranes based on DNA-SWNT and lysozyme-SWNT (where SWNT stands for single-walled carbon nanotubes) were shown to exhibit long-term activity against *S. aureus* and *Micrococcus lysodeikticus*, however only when lysozyme is found on the outermost layer [41]. In our study, the effect of the outermost layer is minimal, given that both (Naf/Lys)_3_ and (Naf/Lys)_3.5_ coated crystals (with lysozyme at pH = 9) exhibit identical antimicrobial performance (Figure S2). Regarding the (Naf/Lys)_6_, it appears that strong Nafion-protein electrostatic interactions keep lysozyme firmly fixed to the LbL assembly without compromising its secondary structure and while allowing its active sites to remain accessible by the bacteria.

The antimicrobial performance of chitosan critically depends upon intrinsic characteristics such as molecular weight and the degree of deacetylation and charge density, as well as external properties such as pH (enhanced activity at low pH), temperature (enhanced activity at higher temperatures) and ionic strength [42]. In general, three modes of action have been identified for chitosan: One arising from its polycationic nature that facilitates lysis of the microbial cell membranes, the second associated with its strong metal-chelating properties that deprives bacterial cells of essential nutrients, and the third stemming from its DNA-binding ability that inhibits protein and mRNA synthesis from the bacterial cells [14].

In analogy to lysozyme, a number of LbL antimicrobial membranes rely on the electrostatic immobilisation of chitosan. To that end, it has been reported that LbL assemblies based on chitosan/hyaluronic acid on PET show excellent activity against *E. coli* [43]. LbL assemblies based on chitosan/polyanionic lentinan sulphate on polyurethane showed 58% improvement against the opportunistic pathogen *P. aeruginosa* [44]. Moreover, LbL assemblies of chitosan and alginates on cotton samples were effective against *S. aureus* and *Klebsiella pneumonia* [45], while chitosan/lignosulphonates multilayers on cellulose fibres suppress *E. coli* growth up to 97% [46].

By comparison, only a limited body of work is centred around the antimicrobial activity of Nafion, even though Nafion coated stainless steel disks were shown to inhibit *E. coli* adhesion [22]. It is widely accepted that repulsive forces between the negatively charged Nafion and the similarly charged bacteria gives rise to a bacterial exclusion zone (EZ) at the Nafion-water interface. A recent study employed confocal laser scanning microscope to show that the EZ is a non-equilibrium phenomenon that diminishes with time, as van der Waals and acid-base forces start to dominate the Nafion-bacteria interactions [17]. Because of those forces, a significant number of cells are able to break the EZ barrier after 48 h of incubation, compared to rare and sporadic cell attachment after 24 h of incubation.

Interestingly, ζ for (Naf/Lys)_6_, (Naf/Chi)_6_, and (Naf/Lys/Naf/Chi)_2_ deposited on the aluminium foil was found +2.8 mV, −22.2 mV, and −30.7 mV, respectively, compared to −52.1 mV for the Nafion coated aluminium foil surface and similar trends were recorded for the multilayers deposited on a polypropylene substrate. Although, Nafion’s negative charges have been overcompensated in (Naf/Lys)_6_ coatings, they exhibit exceptional antimicrobial behaviour that cannot be attributed solely to EZ effects. In that sense, our work demonstrates for the first time that Nafion based compositions exhibit supreme antimicrobial behaviour that goes beyond the concept of the EZ.

## 5. Conclusions

In conclusion, we report a systematic study on the structure-property relationships of a new series of antimicrobial coatings comprising Nafion, chitosan and lysozyme. The chemical composition, the topological characteristics and the wetting performance, are all important parameters that define the superior antimicrobial behaviour of the ultrathin films. Our study provides solid evidence that coupling between Nafion and conventional antimicrobial agents can generate highly effective platform coatings to combat the colonization and spread of bacteria.

## Supplementary Materials

The following are available online at https://www.mdpi.com/2079-4991/9/11/1563/s1, Figure S1: (i) Photos of UV-vis polystyrene cuvettes: Uncoated (A), (Naf/Lys)_6_ (B), (Naf/Chi)_6_ (C), (Naf/Lys/Naf/Chi)_2_ (D), demonstrating their transparency levels. (ii) UV-vis spectra of polystyrene cuvettes coated with (Naf/Lys)_6_, (Naf/Chi)_6_, and (Naf/Lys/Naf/Chi)_2_. Figure S2: Reduction of the population of E. coli and S. aureus cultures exposed to QCM-D crystals coated with (Naf/Lys)_6_ (pH = 4), (Naf/Lys)_6_ (pH = 9), (Naf/Lys)_3_ (pH = 9), (Naf/Lys)_3.5_ (pH = 9), (Naf/Lys/Naf/Chi)_1_, and (Naf/Chi)_3_.
