# Supplementary material for: Layer by Layer Antimicrobial Coatings Based on Nafion, Lysozyme, and Chitosan
Source: Nanomaterials (Basel). 2019 Nov 4;9(11):1563. doi: 10.3390/nano9111563 (PMC6915488; doi:10.3390/nano9111563)
Supplement: Supplementary file 1 [file nanomaterials-09-01563-s001.pdf]

Layer by Layer Antimicrobial Coatings Based on Nafion, Lysozyme and Chitosan

Ella N. Gibbons [1], Charis Winder [2], Elliot Barron [2], Diogo Fernandes [3], Marta J. Krysmann [1,*], Antonios Kelarakis [2,*], Adam V.S. Parry [4], Stephen G. Yeates [4]

[1] School of Pharmacy and Biomedical Sciences, University of Central Lancashire, Preston PR12HE, U.K.
[2] UCLan Research Centre for Smart Materials, School of Physical Sciences and Computing, University of Central Lancashire, Preston PR12HE, U.K.
[3] Malvern Panalytical, Grovewood Road, Enigma Business Park, Malvern, Worcestershire WR14 1XZ, UK
[4] School of Chemistry, University of Manchester, Manchester M13 9PL.
* Correspondence: akelarakis@uclan.ac.uk; mkrysmann@uclan.ac.uk

i

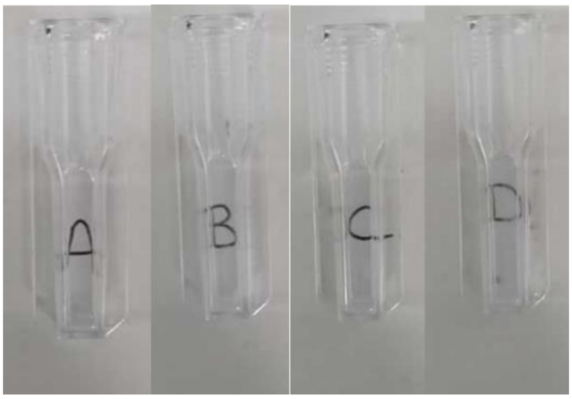

ii

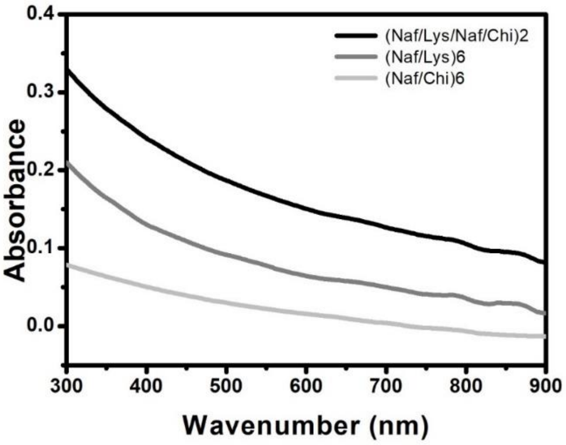

Figure S1. i) Photos of UV-vis polystyrene cuvettes: uncoated (A), $(Naf/Lys)_6$ (B), $(Naf/Chi)_6$ (C), $(Naf/Lys/Naf/Chi)_2$ (D), demonstrating their transparency levels. ii) UV-vis spectra of polystyrene cuvettes coated with $(Naf/Lys)_6$, $(Naf/Chi)_6$, and $(Naf/Lys/Naf/Chi)_2$.

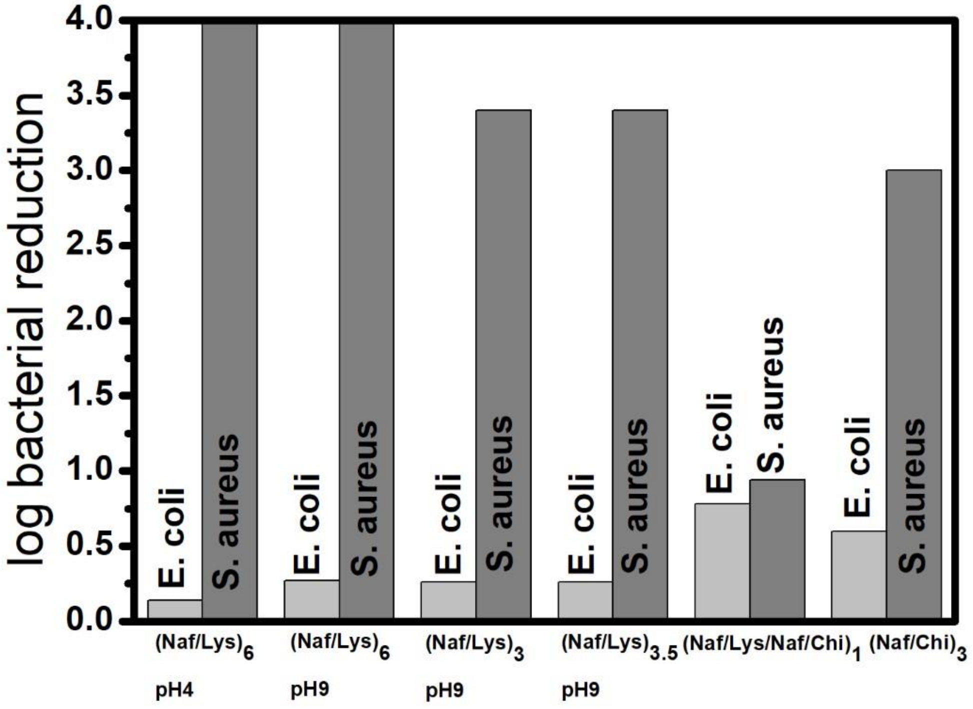

Figure S2. Reduction of the population of *E-coli* and *S. aureus* cultures exposed to QCM-D crystals coated with $(Naf/Lys)_6$ (pH=4), $(Naf/Lys)_6$ (pH=9), $(Naf/Lys)_3$ (pH=9), $(Naf/Lys)_{3.5}$ (pH=9), $(Naf/Lys/Naf/Chi)_1$ and $(Naf/Chi)_3$.
